# Information-Theoretic Analysis of Selected Water Force Fields: From Molecular Clusters to Bulk Properties

**DOI:** 10.3390/e27101073

**Published:** 2025-10-15

**Authors:** Rodolfo O. Esquivel, Hazel Vázquez-Hernández, Alexander Pérez de La Luz

**Affiliations:** 1 Departamento de Química, Universidad Autónoma Metropolitana, Mexico City 09340, Mexico; hazeluh@gmail.com (H.V.-H.); albert_pd@hotmail.com (A.P.d.L.L.); 2 Instituto Carlos I de Física Teórica y Computacional, Universidad de Granada, 18071 Granada, Spain

**Keywords:** water models, information theory, Shannon entropy, Fisher information, molecular dynamics, force fields, complexity measures, cluster analysis, bulk properties

## Abstract

We present a comprehensive information-theoretic evaluation of three widely used rigid water models (TIP3P, SPC, and SPC/ε) through systematic analysis of water clusters ranging from single molecules to 11-molecule aggregates. Five fundamental descriptors—Shannon entropy, Fisher information, disequilibrium, LMC complexity, and Fisher–Shannon complexity—were calculated in both position and momentum spaces to quantify electronic delocalizability, localization, uniformity, and structural sophistication. Clusters containing 1, 3, 5, 7, 9, and 11 molecules (denoted 1 M, 3 M, 5 M, 7 M, 9 M, and 11 M) were selected to balance computational tractability with representative scaling behavior. Molecular dynamics simulations validated the force fields against experimental bulk properties (density, dielectric constant, self-diffusion coefficient), while statistical analysis using Shapiro–Wilk normality tests and Student’s *t*-tests ensured robust discrimination between models. Our results reveal distinct scaling behaviors that correlate with experimental accuracy: SPC/ε demonstrates superior electronic structure representation with optimal entropy–information balance and enhanced complexity measures, while TIP3P shows excessive localization and reduced complexity that worsen with increasing cluster size. The transferability from clusters to bulk properties is established through systematic convergence of information-theoretic measures toward bulk-like behavior. The methodology establishes information-theoretic analysis as a useful tool for comprehensive force field evaluation.

## 1. Introduction

Classical molecular dynamics (MD) simulations of liquids have advanced significantly through the development of force fields that offer an increasingly favorable balance between accuracy and computational efficiency, particularly in the case of nonpolarizable water models. A key milestone in this evolution was the introduction of transferable three- and four-site water models, such as TIP3P, SPC, and TIP4P, which successfully reproduced fundamental physicochemical properties, including liquid density, heat of vaporization, and radial distribution functions, at a moderate computational cost [1].

The development of these models represents a significant achievement in computational chemistry, as water is arguably the most important solvent in chemical and biological systems. Among the three-site models, TIP3P (Transferable Intermolecular Potential with 3 Points) and SPC (Simple Point Charge) have become widely adopted standards. The TIP3P model, developed by Jorgensen and colleagues, employs specific O-H bond lengths (0.9572 Å) and H-O-H angles (104.52°) with optimized partial charges to reproduce liquid water properties [1]. In contrast, the SPC model uses slightly different geometric parameters (O-H bond length of 1.0 Å and H-O-H angle of 109.45°) along with different charge distributions [2,3]. These seemingly minor differences in parameterization lead to significant variations in predicted properties, particularly for the dielectric constant—a critical property for many applications.

The SPC/ε model represents a more recent refinement that addresses the systematic underestimation of the dielectric constant in the original SPC model. By introducing an empirical self-polarization correction, often referred to as a “missing-energy” term, and optimizing the charge distribution specifically to match the experimental static dielectric constant (78.4 at 298 K), the SPC/ε model achieves improved thermodynamic and dielectric behavior while preserving computational simplicity [4,5]. This targeted optimization strategy has proven successful in extending the applicability of SPC-type models to confined and interfacial environments, though the model still does not adequately reproduce the temperature of maximum density—a characteristic anomaly of water [5].

Building on these foundations, a property-driven workflow now connects the customized development of force fields with targeted MD simulations of complex fluids and interfacial systems. This approach emphasizes the systematic calibration of intermolecular parameters against experimentally accessible observables, thereby ensuring that the resulting force fields not only reproduce bulk thermodynamic properties but also extend reliably to heterogeneous environments. Pioneering studies were instrumental in establishing robust protocols for modeling liquid–vapor coexistence, providing accurate predictions of orthobaric densities and surface tensions [6].

The continuous evolution of water model development has proceeded through increasingly sophisticated parametrization strategies. The four-site TIP4P potential represented a significant step forward, as it was the first nonpolarizable model capable of simultaneously reproducing both the dielectric constant and the temperature of maximum density [6]. Building on this success, the TIP4P/ε model restructured the original TIP4P geometry around the same dielectric target, yielding a computationally efficient force field that has been widely adopted for simulations of confined and interfacial water [7]. The flexible FBA/ε potential further extended this benchmark-based strategy by introducing bond stretching and angle bending flexibility while retaining a dielectric-based parametrization scheme, enabling accurate predictions across a broad range of temperatures, pressures, and complex heterogeneous environments [8].

Beyond water modeling, these methodological advances have enabled the development of improved force fields for technologically relevant systems [9,10,11]. Notable examples include united-atom force fields for imidazolium-based room-temperature ionic liquids that reproduce densities, heats of vaporization, and viscosities without explicit polarization [12], and molecular dynamics simulations of propylene carbonate electrolytes containing LiTFSI, LiPF_6_, and LiBF_4_ that successfully map concentration-dependent transport properties in quantitative agreement with experimental data [13].

### 1.1. Information Theory in Molecular Systems

Parallel to these atomistic model developments, information theory has emerged as a complementary framework for quantifying molecular structure, bonding, and reactivity. Within this perspective, concepts such as Shannon entropy, Fisher information, disequilibrium, and statistical complexity indices provide rigorous measures of order, dispersion, and internal coupling that are derived directly from electronic probability distributions and remain independent of the basis set. These information-theoretic descriptors have proven particularly useful for identifying patterns of electron delocalization, detecting signatures of correlation and localization, and establishing quantitative links between electronic structure and chemical observables.

The application of information-theoretic measures to chemical systems has revealed fundamental relationships between electronic structure and molecular properties. Plotting entropy against disequilibrium in “information planes” distinguishes molecular shapes, bonding motifs, and polarity, while complexity indices trace the stepwise enrichment of structures from simple diatomics to essential amino acids [14,15]. Extensions to momentum space and three-dimensional information densities fingerprint equilibrium conformations and monitor bond breaking and formation along reaction coordinates [16]. Fisher information profiles and entropy-type indicators locate the localization–delocalization crossover that defines transition states, offering a phenomenological view that parallels—but remains independent of—potential-energy barriers [17]. Recent applications to proton-transfer equilibria in citric acid illustrate the framework’s capacity to rationalize both kinetic and thermodynamic facets of chemical reactivity [18].

The power of information-theoretic analysis extends to molecular classification schemes. The Predominant Information-Quality Scheme (PIQS) ranks six global descriptors—position and momentum space entropies, Fisher information, and Onicescu disequilibria—within each molecule; the descriptor with the highest normalized value becomes the molecule’s one-letter label, cleanly separating aliphatic, aromatic, polar, and charged amino-acid families [19]. At the trajectory level, mutual information maps extracted from MD ensembles now pinpoint allosteric communication pathways in proteins with residue-level resolution [20], while transfer-entropy calculations reveal the direction of signal propagation, predicting dynamic hotspots in biomolecular networks [21].

Recent developments have further expanded the scope of information-theoretic applications. Single-trajectory entropy estimators close the thermodynamic loop for phase-change simulations, yielding force field-agnostic entropy profiles for liquids and solids [22]. Coarse-graining schemes that maximize mutual information between atomistic and reduced representations generate transferable mesoscopic force fields while preserving key dynamical correlations [23], and information-content metrics provide objective criteria for trajectory completeness and uncertainty assessment [24].

### 1.2. Objectives and Scope

This work presents a systematic information-theoretic analysis of water clusters generated using three widely employed rigid water models: TIP3P, SPC, and SPC/ε. Our primary objective is to establish quantitative relationships between force field parameters, information-theoretic descriptors, and experimentally observable properties. By analyzing clusters ranging from single molecules to 11-molecule aggregates (denoted as 1 M, 3 M, 5 M, 7 M, 9 M, and 11 M), we capture essential scaling behaviors.

This manuscript is organized as follows: Section 2 details our computational methodology, including force field parameters, simulation protocols, and the calculation of electronic densities using density functional theory. Section 3 provides the theoretical framework for our information-theoretic measures, explaining their physical significance and expected behaviors. Section 4 describes our statistical validation methods, including normality testing and mean comparison procedures. Section 5 presents our results, demonstrating how information-theoretic measures evolve with cluster size and correlate with bulk properties. Finally, Section 6 discusses the implications for force field selection and water modeling, establishing clear connections between cluster-level analysis and bulk water behavior.

## 2. Computational Methodology and Validation Studies

To demonstrate the practical application of information-theoretic analysis for force field evaluation, we present a detailed comparative study of three widely used rigid water models and their ability to reproduce key physicochemical properties across multiple length scales.

### 2.1. Force Field Parameters and Simulation Details

Three rigid water models were employed for molecular dynamics simulations: TIP3P [1], SPC [3,4], and SPC/ε [5]. These models differ in their geometric parameters and charge distributions, as detailed in Table 1. During all simulations, the molecular geometries were constrained to remain rigid using the LINCS algorithm [25], maintaining the bond lengths and angles specified for each force field. This constraint ensures that the differences observed in our information-theoretic analysis arise from the distinct parameterizations and their effects on intermolecular interactions rather than from intramolecular flexibility.

The functional form for nonbonding interactions in these water models combines Lennard-Jones and Coulomb terms:

(1) $$V\left(r_{ij}\right)=4\varepsilon_{OO}\left[{\left(\frac{\sigma_{OO}}{r_{ij}}\right)}^{12}-{\left(\frac{\sigma_{OO}}{r_{ij}}\right)}^{6}\right]+\frac{q_{i}q_{j}}{4\pi \varepsilon_{0}r_{ij}},$$

where $r_{ij}$ is the distance between sites *i* and *j*, $\varepsilon_{OO}$ is the Lennard-Jones energy parameter, $\sigma_{OO}$ is the diameter for an O–O pair, $q_{i}$ and $q_{j}$ are the electric charges of sites *i* and *j*, and $\varepsilon_{0}$ is the permittivity of a vacuum. Cross-interactions were calculated using Lorentz–Berthelot mixing rules: $\sigma_{ij}=\left(\sigma_{ii}+\sigma_{jj}\right)/2$ and $\varepsilon_{ij}={\left(\varepsilon_{ii}\varepsilon_{jj}\right)}^{1/2}$.

### 2.2. Molecular Dynamics Simulation Protocol

Molecular simulations were performed at 298.15 K and 1 bar to calculate bulk physicochemical properties. Liquid simulations employed 1000 water molecules in the *NPT* ensemble (constant number of particles, pressure, and temperature). Molecular dynamics simulations were conducted using GROMACS [26]. The equations of motion were integrated using the leap-frog algorithm [27] with a time step of 2 fs and periodic boundary conditions in all three directions.

Electrostatic interactions were calculated using the particle mesh Ewald approach [28] with a tolerance of $10^{-6}$ for the real-space contribution, grid spacing of 1.2 Å, and fourth-order spline interpolation for reciprocal space. Bond distances and angles were constrained to their equilibrium values using the LINCS algorithm to maintain rigid molecular geometries throughout the simulations. Temperature coupling employed the Nosé–Hoover thermostat with $\tau_{T}=0.5$ ps, while pressure coupling used the Parrinello–Rahman barostat with $\tau_{P}=1.0$ ps. These parameters were chosen to adequately sample volume fluctuations [29].

The dielectric constant was calculated from dipole moment fluctuations [30,31], and liquid properties were averaged over at least 100 ns following a 20 ns equilibration period. Table 2 summarizes the calculated physicochemical properties for the three water models at 298.15 K and 1 bar.

The results demonstrate that SPC/ε provides the most accurate reproduction of experimental density and the dielectric constant, validating the design philosophy of optimizing charge distribution around the experimental dielectric benchmark. TIP3P significantly overestimates both the dielectric constant and self-diffusion coefficient, while SPC underestimates the dielectric constant but provides reasonable density values.

### 2.3. Water Cluster Identification and Analysis

During molecular simulations, water clusters of varying sizes were identified using the Sevick method [36], which defines clusters based on oxygen–oxygen distance criteria. Atomic positions were saved every 10 steps to track water cluster trajectories. Initial cluster searches performed every 500 steps resulted in lost trajectories; reducing the interval to every 100 steps enabled successful cluster tracking.

The notation used throughout this manuscript (1 M, 3 M, 5 M, 7 M, 9 M, 11 M) refers to clusters containing 1, 3, 5, 7, 9, and 11 water molecules, respectively. We selected odd-numbered cluster sizes to capture essential scaling behaviors while maintaining computational efficiency. This selection allows us to observe the progressive development of hydrogen bonding networks from isolated molecules to bulk-like aggregates without the prohibitive computational cost of analyzing every possible cluster size. The experimental coordination number of 4.7 water molecules in the first solvation shell [37] provides a benchmark for evaluating the structural accuracy of different force fields.

**Figure 1:**
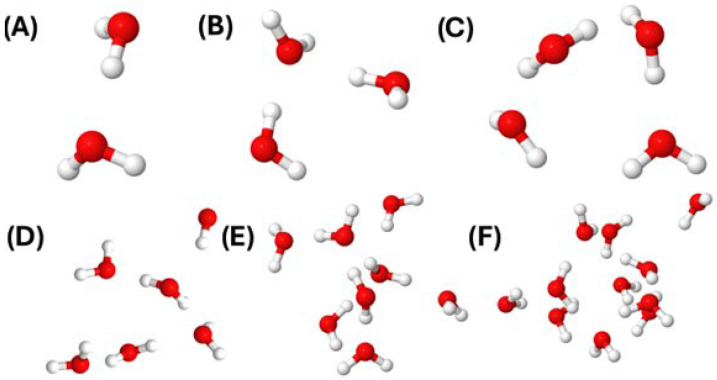
Representative water clusters identified using the Sevick method during molecular dynamics simulations at 298.15 K and 1 bar. The clusters shown contain (**A**) 2 molecules, (**B**) 3 molecules, (**C**) 4 molecules, (**D**) 5 molecules, (**E**) 7 molecules, and (**F**) 11 molecules. Oxygen atoms are colored red and hydrogen atoms are white. The progression from small to large clusters illustrates the development of increasingly complex hydrogen bonding networks, with larger clusters beginning to exhibit bulk-like structural features.

Figure 1 illustrates representative water clusters obtained from the simulations, showing the progression from dimers to 11-molecule aggregates. The molecular arrangements demonstrate the characteristic hydrogen bonding patterns that emerge at different cluster sizes, with oxygen atoms shown in red and hydrogen atoms in white.

### 2.4. Electronic Structure Calculations

The electron densities of the water clusters were obtained using density functional theory (DFT) calculations performed with the Gaussian 09 software package [38]. All computations employed the M06-2X exchange–correlation functional with the 6-311++G(d,p) basis set. The molecular geometries were kept fixed at the force field equilibrium values to ensure that the information-theoretic analysis reflects the electronic structure corresponding to each specific force field parameterization. An ultrafine integration grid was employed to ensure numerical accuracy in the density calculations.

The M06-2X functional was selected based on its demonstrated performance in benchmark studies for calculating non-covalent interaction energies and thermochemical properties [39]. While more accurate functionals exist, we considered M06-2X adequate for the current study, as our primary goal is to demonstrate the applicability of information-theoretic measures for classifying and evaluating classical force fields rather than obtaining highly precise absolute numerical values. The consistency of using the same functional across all force fields ensures that relative comparisons remain valid and meaningful. Although benchmarking these measures is a necessary next step, it is beyond the scope of this work.

The calculated electron densities were subsequently used to compute information-theoretic measures in both position and momentum spaces, as detailed in the following section. This approach allows us to establish quantitative relationships between force field parameters, electronic structure characteristics, and macroscopic properties.

## 3. Information-Theoretic Measures

Under the independent-particle framework, molecular systems may be described using their electronic density profiles in both position (*r*-space) and momentum (*p*-space) representations. These complementary representations provide distinct but related perspectives on electronic structure, each offering unique insights into molecular properties and behaviors.

### 3.1. Electronic Density Representations

The position space total electron density $\rho (\vec{r})$ is constructed from molecular position space orbitals $\psi_{i}\left(\vec{r}\right)$, while the momentum space density $\gamma (\vec{p})$ is built from molecular momentals (momentum space orbitals) $\phi_{i}\left(\vec{p}\right)$. The momentals are derived through three-dimensional Fourier transformation of their position space counterparts:

(2) $$\phi_{i}\left(\vec{p}\right)={\left(2\pi \right)}^{-3/2}\int \exp\left(-i\vec{p}\;\cdot \;\vec{r}\right)\psi_{i}\left(\vec{r}\right)d\vec{r}.$$

Atomic units are utilized throughout this work. Established methodologies for Fourier transforming position space orbitals produced by ab initio calculations have been documented [40]. Since ab initio orbitals are expressed as linear combinations of atomic basis functions, and analytical Fourier transforms of these basis functions are available [41], the conversion of complete molecular electronic wavefunctions from position to momentum space is computationally accessible [42].

### 3.2. Shannon Entropy

The Shannon entropy *S* for a probability density quantifies the overall electronic dispersion within the molecular configuration space, serving as an indicator of electron density delocalization. For a unit-normalized probability density in position space, it is expressed through the logarithmic functional [43]:

(3) $$S\left[\rho \right]=-\int \rho \left(\vec{r}\right)\ln\rho \left(\vec{r}\right)d\vec{r}.$$

This parameter reaches its maximum value when information about $\rho (\vec{r})$ is minimal and the distribution becomes delocalized. In water clusters, higher Shannon entropy indicates greater electronic delocalization, reflecting enhanced charge mobility that facilitates hydrogen bonding and polarization effects. The expected behavior shows entropy increasing with cluster size as electronic distributions become more diffuse through hydrogen bonding network formation.

### 3.3. Fisher Information

Unlike the global Shannon entropy, Fisher information *I* exhibits local characteristics due to its high sensitivity to rapid distribution changes within confined regions. This quantity is defined by the gradient-density functional [44,45]:

(4) $$I\left[\rho \right]=\int \frac{|\vec{\nabla }\rho \left(\vec{r}\right){|}^{2}}{\rho (\vec{r})}d\vec{r}.$$

Fisher information evaluates the gradient characteristics of the electron distribution, assessing the spatial pointwise concentration of the electronic probability cloud. In water systems, it reflects the balance between covalent bonding (high localization) and hydrogen bonding (delocalization effects). Higher values indicate sharper, more localized electronic features with steep gradients. The expected trend shows Fisher information generally decreasing with cluster size as hydrogen bonding creates more diffuse electronic environments.

### 3.4. Disequilibrium

The disequilibrium *D*, also known as self-similarity [46] or information energy [47], measures the deviation from uniformity in the probability density. For position space, it is expressed as

(5) $$D\left[\rho \right]=\int \rho^{2}\left(\vec{r}\right)d\vec{r}.$$

This measure captures the anisotropy inherent in hydrogen-bonded systems, reflecting the directional nature of hydrogen bonding and molecular orientation effects. Higher disequilibrium indicates greater non-uniformity in charge distribution. The evolution with cluster size shows complex behavior, initially increasing with cluster formation as hydrogen bonding creates charge asymmetries, then potentially stabilizing as bulk-like patterns emerge.

### 3.5. Complexity Measures

Beyond individual entropic measures, quantifying physical system complexity provides additional insights into structural organization. We employ two complementary complexity measures that combine different information-theoretic quantities.

The López-Ruiz–Mancini–Calbet (LMC) complexity measure [48,49] combines disequilibrium with Shannon exponential entropy:

(6) $$C_{LMC}=D\left[\rho \right]\;\cdot \;e^{S[\rho ]}.$$

This measure captures the balance between organization (disequilibrium) and disorder (entropy), reflecting the sophisticated structural arrangements characteristic of hydrogen-bonded water networks. The parameter satisfies the constraint $C_{LMC}\geq 1$ for any three-dimensional probability density.

The Fisher–Shannon (FS) complexity measure [50,51] combines local and global characteristics:

(7) $$C_{FS}=I\left[\rho \right]\;\cdot \;J\left[\rho \right],$$

where $J\left[\rho \right]=\frac{1}{2\pi e}e^{\frac{2}{3}S\left[\rho \right]}$ represents the Shannon power entropy. This parameter quantifies the coexistence of localized (covalent) and delocalized (hydrogen bonding) electronic features, capturing the dual nature of water’s electronic structure. It satisfies the lower bound $C_{FS}\geq 3$ for any three-dimensional probability density.

These complexity measures are dimensionless, invariant under replication, translation, and scaling transformations [52], and exhibit minimum values for extreme probability distributions (perfect order and maximum disorder). In water clusters, complexity should increase with size as more sophisticated hydrogen bonding patterns develop, reaching maximum values for optimal structural organization.

All measures presented above can be directly extended to momentum space using the corresponding momentum density $\gamma (\vec{p})$, providing complementary information about kinetic energy distributions and nuclear motion effects.

All calculated values for the information-theoretic (IT) measures are available in the accompanying *.csv* files from the Supplementary Materials. Each file is labeled according to the following order: number of water molecules, integration space (position or momentum), and force field name. The files include values for each sampled time step.

## 4. Statistical Method: Student’s *t*-Test

To rigorously assess whether the mean values of information-theoretic measures differ significantly between force fields, we employ comprehensive statistical analysis combining normality testing and mean comparison procedures.

### 4.1. Shapiro–Wilk Test for Normality

Before applying parametric tests, we verify that our data follows a normal distribution using the Shapiro–Wilk test [53], which is particularly suitable for small samples (n<50). The test statistic is given by

(8) $$W=\frac{{\left(\sum_{i=1}^{n}a_{i}x_{\left(i\right)}\right)}^{2}}{\sum_{i=1}^{n}{\left(x_{i}-\bar{x}\right)}^{2}},$$

where $x_{i}$ is the *i*th value in the sample, $x_{\left(i\right)}$ is the *i*th smallest value, $\bar{x}$ is the sample mean, and $a_{i}$ are coefficients obtained from a standard normal distribution. The null hypothesis is that the sample is drawn from a normal distribution. If the *p*-value exceeds 0.05, we fail to reject the null hypothesis, indicating that the data distribution may be normal.

### 4.2. Probability Plots

To further confirm normality, we compare our data against standard normal distributions through probability plots. Theoretical quantiles $Q_{i}$ for the normal distribution are computed from the probability estimates proposed by Filliben [54]:

(9) $$p_{i}=\left\{\begin{array}{cc} 0.5^{1/n} & i=1\\ \left(i-0.3175)/(n+0.365\right) & i\in [2,n-1]\\ 1-0.5^{1/n} & i=n \end{array},\right.$$

where *n* is the number of values in the dataset. When the coefficient of determination ($R^{2}$) exceeds 0.9 and the Shapiro–Wilk *p*-value is greater than 0.05, we consider these as strong statistical evidence supporting the normality of the data distribution.

### 4.3. Welch’s t-Test

Once normality is confirmed, we compare force fields pairwise using Welch’s *t*-test [55], which does not assume equal variances between samples. The test statistic is

(10) $$t=\frac{{\bar{X}}_{1}-{\bar{X}}_{2}}{\sqrt{s_{{\bar{X}}_{1}}^{2}+s_{{\bar{X}}_{2}}^{2}}},$$

where ${\bar{X}}_{i}$ is the mean, $s_{{\bar{X}}_{i}}=s_{i}/\sqrt{N_{i}}$ is the standard error, $s_{i}$ is the standard deviation, and $N_{i}$ is the size of the *i*th sample. The degrees of freedom are approximated using the Welch–Satterthwaite equation [56]. For the *t*-test, the null hypothesis is that the means of the distributions are equal. If the calculated *p*-value is lower than 0.05, we reject the null hypothesis, meaning that the means of the samples are significantly different.

All statistical tests were performed using the SciPy Python library [57].

## 5. Results and Discussion

Our comprehensive information-theoretic analysis reveals fundamental differences between the three water force fields that correlate strongly with their ability to reproduce experimental properties. The analysis progresses from single molecules to 11-molecule clusters, demonstrating how electronic structure representations scale toward bulk behavior.

### 5.1. Single-Molecule Analysis: Geometric Effects

The information-theoretic analysis of single water molecules (1 M) primarily reflects the geometric differences between force fields. Since the geometries of the water molecules were kept fixed during the calculations, only subtle geometric variations were detected. These variations are likely due to the algorithm used to maintain rigid geometries and numerical precision during the integrations. Consequently, the SPC and SPC/ε force fields, which employ identical water geometries (O-H bond length of 1.0 Å and H-O-H angle of 109.45°), resulted in almost identical distributions and close mean values. In contrast, TIP3P’s distinct geometry (O-H bond length of 0.9572 Å and H-O-H angle of 104.52°) yields significantly different values across all measures. Thus, this first part of the discussion focuses on how the IT-measures reveal the characteristics of the water models’ geometries.

**Figure 2:**
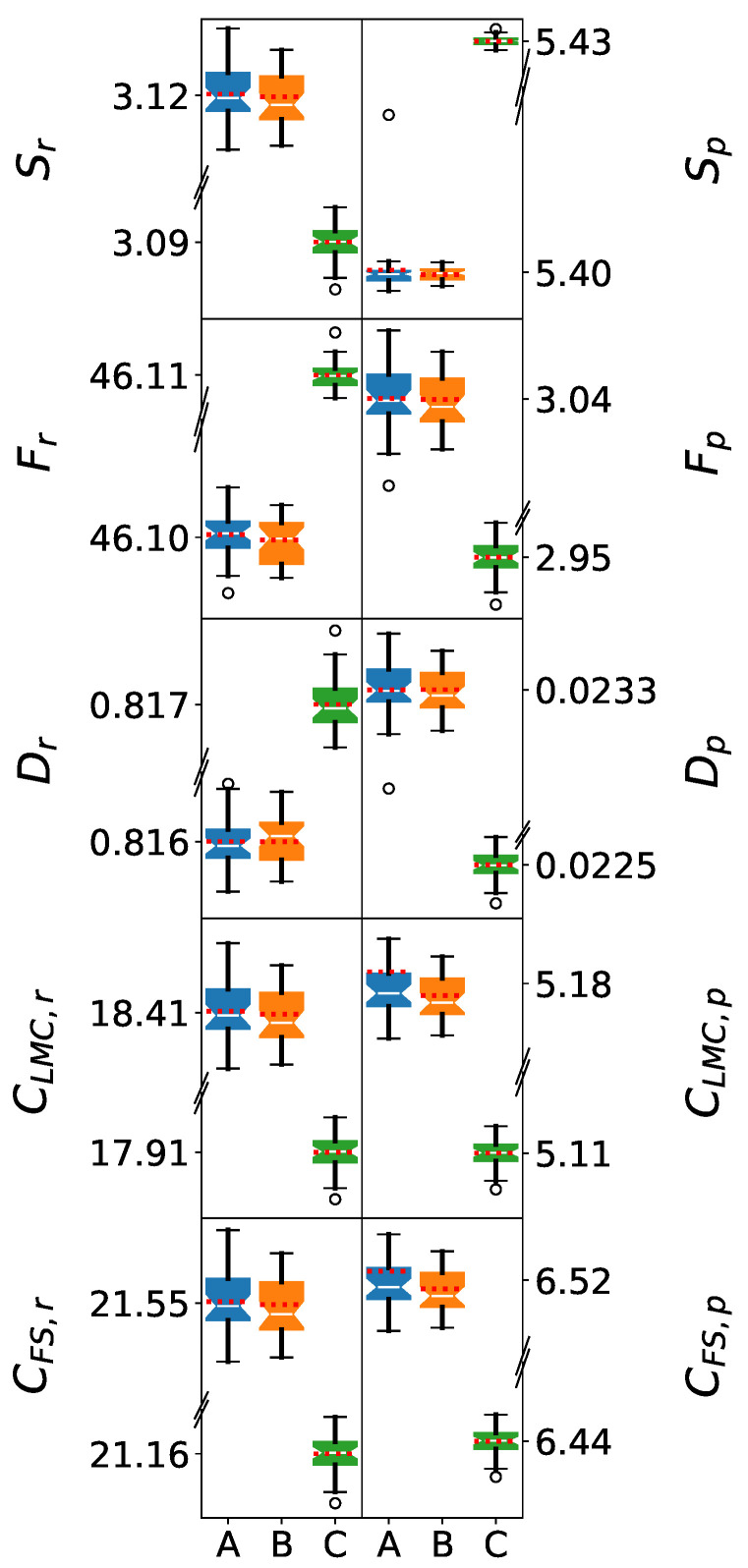
Box plots of information-theoretic measures in position (**left**) and momentum (**right**) spaces for single water molecules. The models shown are (A) SPC, (B) SPC/ε, and (C) TIP3P. The whiskers denote the lowest and highest values, while the bottom, notch, and top of the boxes correspond to the first, second (median), and third quartiles, respectively. Outliers, defined as values lying more than 1.5 times the interquartile range below the first quartile or above the third quartile, are represented by open circles. The mean value for each dataset is indicated by a red dotted line. For clarity, the y-axes are discontinuous, as the data ranges for $C_{LMC,r}$ and $C_{FS,r}$ are on the order of $10^{-2}$ a.u., while the remaining descriptors are on the order of $10^{-3}$ a.u.

Figure 2 presents box plots of the information-theoretic measures for single molecules. The normality of the distributions can be visually assessed by the near-symmetric boxes, where the mean values (red dotted lines) closely align with the medians (notches). This normality is crucial for the reliable application of Welch’s *t*-tests in comparing force field performance. Additionally, Welch’s *t*-tests revealed that the differences between the SPC and SPC/ε force fields are not statistically significant. The box plots for these models appear similar, with those corresponding to SPC/ε exhibiting slightly narrower distributions. The mean values are closely aligned for these models, demonstrating that there is no clear statistical separation between the distributions obtained from these two force fields.

The significant differences observed in the IT-measures between the SPC force fields and TIP3P are mainly related to the geometries of the water models. The single-molecule statistics underscore that the primary differences among the three models for the water clusters will be related to their underlying molecular geometries. Any additional discrepancies are thus attributable to how each force field simulates intermolecular interactions, as will be discussed in the next sections.

### 5.2. Five-Molecule Clusters: Emergence of Intermolecular Effects

The analysis of 5 M clusters reveals how intermolecular interactions differentiate the force fields beyond geometric effects. At this cluster size, hydrogen bonding networks begin to establish characteristic patterns that distinguish the models’ electronic structure representations.

**Figure 3:**
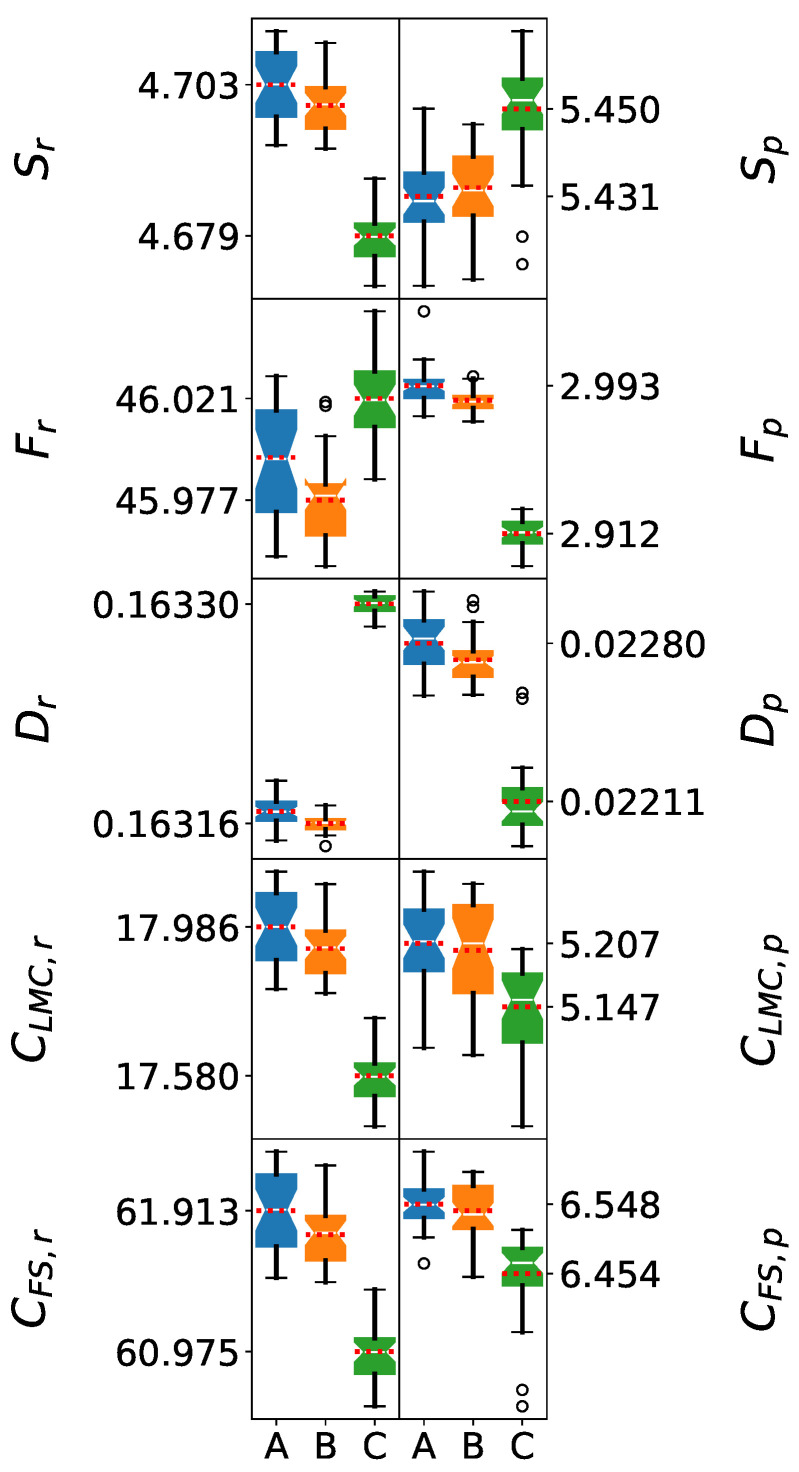
Box plots of information-theoretic measures for 5-molecule water clusters. The models shown are (A) SPC, (B) SPC/ε, and (C) TIP3P. The whiskers denote the lowest and highest values, while the bottom, notch, and top of the boxes correspond to the first, second (median), and third quartiles, respectively. Outliers, defined as values lying more than 1.5 times the interquartile range below the first quartile or above the third quartile, are represented by open circles. The mean value for each dataset is indicated by a red dotted line.

The Shapiro–Wilk test results demonstrate excellent data quality for the 5 M cluster analysis. Both position and momentum space *p*-values consistently exceed the 0.05 significance threshold across all information-theoretic measures and force fields. The boxes of each distribution shown in Figure 3 are almost symmetric, and the mean values closely align with the medians, confirming the normality of the distributions. The robust normality observed in 5 M clusters suggests that this cluster size provides sufficient statistical sampling while avoiding potential artifacts that might arise in very small (1 M–3 M) or very large (>9 M) clusters.

In addition, the Welch’s *t*-test results demonstrate high statistical significance for most pairwise force field comparisons. In position space, all *p*-values were lower than 0.05, indicating a statistically significant difference between the mean values of each IT-measure. This is evident in Figure 3, where the mean values are clearly separated. This contrasts with the single-molecule analysis, where SPC and SPC/ε values were statistically indistinguishable. The high statistical significance validates the use of information-theoretic measures in position space for quantitative force field classification.

In contrast, in momentum space, the *p*-values for the *t*-test between SPC and SPC/ε were above the 0.05 threshold, with the exception of the $F_{p}$ and $D_{p}$ descriptors. While some differences between the mean values of the IT-measures of these force fields can be visually observed in momentum space (Figure 3), there is not enough statistical evidence to support a clear separation beyond those two descriptors. This suggests that the higher dispersion of the electron densities in momentum space, as opposed to position space, prohibits a clear distinction between the information descriptors of SPC and SPC/ε.

The position space analysis reveals a clear hierarchy in electronic delocalization. SPC/ε exhibits intermediate Shannon entropy values, indicating optimal electronic delocalization consistent with its enhanced dielectric parameterization. This balanced delocalization facilitates more realistic hydrogen bonding and charge transfer effects. The geometry employed in the SPC/ε model corresponds to greater electronic delocalization, consistent with its enhanced dielectric properties. Conversely, SPC shows higher entropy values, indicating excessive electronic dispersion, while TIP3P consistently yields the lowest values, suggesting overly constrained electronic distributions. These extremes may limit their ability to accurately represent water’s polarizable nature.

Fisher information shows analogous trends, with TIP3P exhibiting excessive electronic localization that may result from its simplified charge distribution. SPC/ε demonstrates the most appropriate balance between localization and delocalization, essential for accurate representation of both covalent and hydrogen bonding. The disequilibrium measures reveal subtle but significant differences in charge distribution patterns, with all force fields showing comparable magnitudes but distinct fine structures that affect properties sensitive to charge distribution details.

Complexity analysis provides particularly revealing insights. LMC complexity values rank as SPC > SPC/ε > TIP3P, with SPC/ε demonstrating balanced complexity that correlates with its improved ability to reproduce experimental properties. This balance reflects SPC/ε’s capacity to capture configurational diversity while maintaining a realistic ordering of the hydrogen-bonded networks. Fisher–Shannon complexity shows similar trends, confirming that SPC/ε’s superior performance stems from its more nuanced representation of electronic structure, rather than simply different parameter values.

However, information-theoretic measures in momentum space exhibit very broad distributions. Consequently, not all descriptors were adequate to establish a distinction between the SPC and SPC/ε force fields, with the exception of the $I_{p}$ and $D_{p}$ measures. This highlights the importance of statistical analysis for selecting the most adequate descriptors and ensuring a correct comparison between force fields.

### 5.3. Eleven-Molecule Clusters: Approach to Bulk Behavior

The analysis of 11 M clusters provides crucial insights into force field scalability and the transition toward bulk-like behavior. At this size, hydrogen bonding networks become sufficiently complex to exhibit features characteristic of bulk water.

**Figure 4:**
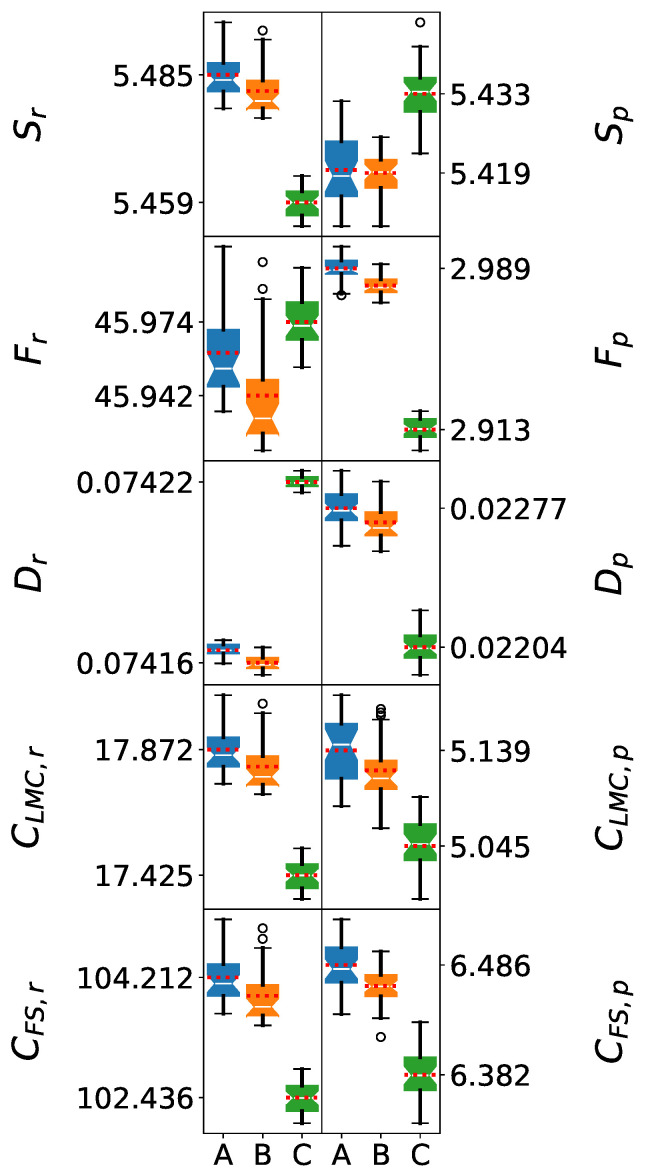
Box plots of information-theoretic measures for 11-molecule water clusters. The models shown are (A) SPC, (B) SPC/ε, and (C) TIP3P. The whiskers denote the lowest and highest values, while the bottom, notch, and top of the boxes correspond to the first, second (median), and third quartiles, respectively. Outliers, defined as values lying more than 1.5 times the interquartile range below the first quartile or above the third quartile, are represented by open circles. The mean value for each dataset is indicated by a red dotted line.

The Shapiro–Wilk test results for 11 M clusters demonstrate a deviation from normality for the information-theoretic measures in position space for the SPC and SPC/ε force fields. The corresponding *p*-values are lower than 0.05, except for $D_{r}$. In addition, the comparison against normal distributions (probability plots) resulted in $R^{2}$ values lower than 0.92, in contrast to those greater than 0.95 for the 5 M clusters. This is evident in Figure 4, where the box plots for the position space are asymmetric and the mean value lines are far from the medians, with the exception of $D_{r}$. These results reveal a limit in the number of molecules that can be considered for a homogeneous description of the systems for these force fields.

In particular, the SPC/ε force field resulted in $R^{2}$ values lower than 0.87. Thus, it exhibited the highest deviations from normality, which suggests a higher diversity in the molecular arrangements generated by this force field. Conversely, in the case of the TIP3P force field, all information-theoretic measures in position space still exhibit normal distributions as in the previous cases.

In contrast, all the information-theoretic measures’ distributions in momentum space are normal. All *p*-values for the Shapiro–Wilk tests are higher than 0.05, and the $R^{2}$ values for the probability plots were higher than 0.96, with the exception of $C_{LMC,p}$ with the SPC/ε force field. This can be observed in Figure 4, where all the box plots are symmetric and the mean values are close to the medians, except for the aforementioned metric and force field. Therefore, the information-theoretic measures in momentum space are unaffected by the inhomogeneities of the clusters in position space.

Despite deviations from normality for the distributions in position space, the Welch’s *t*-test yielded *p*-values below 0.05 for the comparison of the information-theoretic measures of SPC and SPC/ε, indicating statistically significant differences between their mean values. Given Welch’s robustness to unequal variances and moderate non-normality, this result supports a statistically significant difference between these two force fields. Moreover, the relative ordering given by each information-theoretic measure for the aforementioned force fields is maintained for the different cluster sizes.

On the other hand, although the information-theoretic measures were normally distributed in momentum space, the Welch’s *t*-test yielded *p*-values above 0.05 for $S_{p}$ for the SPC and SPC/ε force fields. As a result, a statistical distinction between the mean values of $S_{p}$ cannot be established for the aforementioned force fields.

The amplified differences at 11 M suggest that force field limitations become increasingly problematic as system size approaches bulk limits. SPC/ε maintains optimal electronic delocalization and enhanced complexity measures, demonstrating excellent scalability. While the limited scalability of this force field makes a robust statistical analysis difficult as the clusters deviate from homogeneity, this type of analysis is also useful for revealing the complexity of the molecular networks generated by this force field. TIP3P shows severely excessive localization and significantly reduced complexity, indicating fundamental inadequacies for bulk water modeling. SPC exhibits intermediate performance, characterized by excessive electronic delocalization and structural complexity, which may compromise its accuracy in bulk simulations.

### 5.4. Size-Dependent Evolution: From Clusters to Bulk

**Figure 5:**
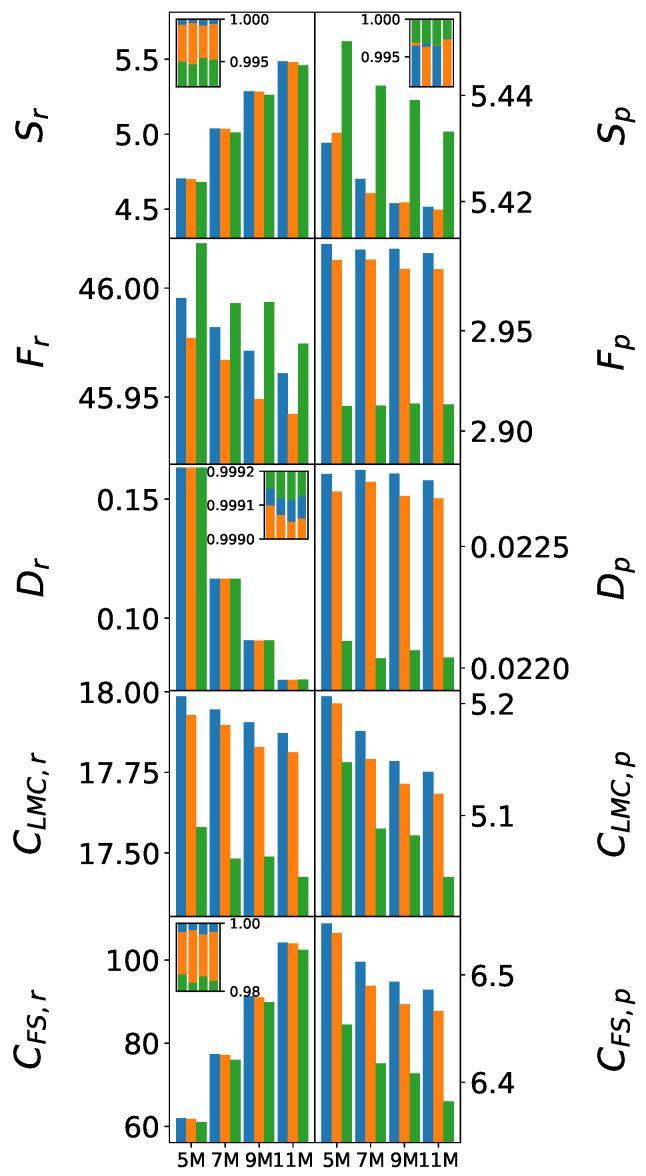
Evolution of information-theoretic measures’ mean values with cluster size in (**left**) and momentum (**right**) spaces. The color scheme follows previous figures: (blue) SPC, (orange) SPC/ε, and (green) TIP3P. The inset plots display normalized values of $S_{r}$, $S_{p}$, $D_{r}$, and $C_{FS,r}$, where the contributions from the three force fields are shown as stacked bars for each cluster size to facilitate relative comparison.

Figure 5 illustrates the systematic evolution of information-theoretic measures with increasing cluster size, highlighting how force field differences scale toward bulk behavior. Only mean values are shown, as the statistical test outcomes for the 7 M and 9 M clusters closely mirror those of the 5 M cluster. For $S_{r}$, $S_{p}$, $D_{r}$, and $C_{FS},r$, the values are additionally normalized and displayed in inset plots to enable adequate visual comparison.

The Shannon entropy scaling reveals that fundamental differences in electronic delocalization are preserved across cluster sizes. All three force fields exhibit a steady, monotonic increase in entropy with cluster size, reflecting progressive electronic delocalization driven by the growing number of molecules. Nonetheless, the relative ordering remains consistent, indicating that the nuances distinguishing the force fields persist across all molecular clusters. This pattern further corroborates that, in the case of SPC/ε, intermediate electronic delocalization is characteristic of accurate water models.

Fisher information evolution shows complementary trends. TIP3P maintains excessively high values with a slow decrease upon cluster growth, suggesting persistent over-localization even in larger clusters. SPC/ε exhibits appropriate scaling, with Fisher information decreasing as hydrogen bonding networks develop and create more diffuse electronic environments.

Complexity measures demonstrate the most dramatic force field discrimination. LMC complexity shows a clear ranking (SPC >> SPC/ε > TIP3P) that amplifies with cluster size. SPC/ε’s complexity values decrease slightly, indicating that its ability to capture the nuanced structure of hydrogen-bonding networks remains robust across scales. Fisher–Shannon complexity confirms these trends, with SPC/ε consistently operating in optimal complexity ranges that balance localized and delocalized electronic features.

The systematic convergence of information-theoretic measures toward characteristic values as cluster size increases establishes the connection between cluster-level analysis and bulk properties. The superior scaling behavior of SPC/ε correlates directly with its accurate reproduction of experimental bulk properties (Table 2), while TIP3P’s poor scaling explains its significant overestimation of the dielectric constant and self-diffusion coefficient.

### 5.5. Physical Interpretation and Implications

The information-theoretic analysis provides quantitative insights into how force field parameterization affects water’s electronic structure representation across multiple length scales. The superior performance of SPC/ε stems from its optimized charge distribution that enables appropriate electronic delocalization and configurational diversity, crucial for water’s anomalous properties. This balance is reflected in the model’s intermediate Shannon entropy values, indicating a structural flexibility tempered by sufficient rigidity to allow for a more realistic reproduction of dynamic hydrogen bonding networks.

These electronic structure differences have direct consequences for macroscopic properties. The enhanced electronic delocalization in SPC/ε facilitates an accurate representation of water’s high dielectric constant through appropriate charge mobility and polarization effects. The balanced Fisher information suggests an optimal representation of both short-range (covalent) and long-range (hydrogen bonding) interactions, essential for transport properties like diffusion. The enhanced complexity measures reflect the model’s ability to capture water’s structural sophistication, correlating with accurate thermodynamic properties.

Shannon entropy is an indicator of the degree of molecular order and disorder in liquids simulated using different force fields. In the SPC/ε model, intermediate entropy values reflect optimal configurational diversity, favoring delocalization and structural flexibility. In contrast, the TIP3P model exhibits lower entropies, associated with constrained configurations and less adaptive capacity in complex hydrogen bonding networks. The quality of these hydrogen bonds influences parameters such as the dielectric constant, heat of vaporization, ionic solvation, and reaction dynamics in a solution.

Furthermore, as the system size increases, the differences between models become more pronounced. SPC/ε can more accurately capture collective electronic effects and thus predict macroscopic properties such as surface tension, viscosity, and density.

The scaling analysis demonstrates that force field quality assessment at the cluster level provides reliable predictions of bulk behavior. The systematic evolution of information-theoretic measures from clusters to bulk-like behavior validates the transferability of our conclusions. Force fields showing appropriate scaling of electronic delocalization, balanced localization–delocalization, and increasing structural complexity with system size are most likely to accurately represent bulk water properties.

## 6. Conclusions

This comprehensive information-theoretic investigation of water clusters provides definitive evidence for significant performance disparities between the TIP3P, SPC, and SPC/ε force fields. Through systematic analysis of clusters ranging from single molecules to 11-molecule aggregates, we establish quantitative relationships between force field parameters, electronic structure representations, and macroscopic properties.

The key findings of our study demonstrate that SPC/ε emerges as the superior choice for water simulations across all length scales. Its exceptional performance stems from optimized charge distribution that enables appropriate electronic delocalization, as evidenced by optimal Shannon entropy scaling, balanced Fisher information evolution, and enhanced complexity measures that increase systematically with cluster size. These characteristics translate directly to the accurate reproduction of experimental bulk properties, particularly the dielectric constant and density.

In contrast, TIP3P exhibits fundamental limitations that become increasingly severe with system size. The model’s excessive electronic localization, minimal entropy scaling, and reduced complexity measures indicate inadequate representation of water’s electronic flexibility. These deficiencies manifest as a significant overestimation of the dielectric constant and self-diffusion coefficient, suggesting that TIP3P’s applicability should be restricted to applications where electronic structure accuracy is not critical.

SPC demonstrates intermediate performance that, while superior to TIP3P, shows concerning trends with increasing cluster size. The model provides reasonable approximations for small systems but exhibits scalability limitations. Furthermore, it exhibits excessive electronic delocalization and disordered configurations, leading to deviations from realistic hydrogen-bonded networks. Both aspects may compromise accuracy in bulk simulations requiring precise electronic structure representation.

The methodological contributions of this work extend beyond specific force field comparisons. We establish information-theoretic analysis as a powerful framework for comprehensive force field evaluation, providing objective, quantitative measures of electronic structure quality that complement traditional validation approaches based on thermodynamic and structural properties. The demonstrated correlation between cluster-level information-theoretic descriptors and bulk water properties offers a computationally efficient strategy for force field assessment and development.

The observation that structural diversity increases with cluster size, particularly for SPC/ε, provides insights into the relationship between force field flexibility and realistic water behavior. The deviation from statistical normality in large clusters for the more sophisticated models suggests that capturing water’s full structural complexity may require accepting increased configurational heterogeneity—a feature that simpler models like TIP3P fail to reproduce. Information-theoretic measures deviate from normality, indicating more diverse molecular arrangements as a consequence of the description of intermolecular interactions.

Future directions for this research include extending the analysis to polarizable water models, investigating temperature-dependent effects, and applying the methodology to other molecular systems. The information-theoretic framework developed here could accelerate force field development by providing early-stage quality metrics before extensive bulk simulations. Integration with machine learning approaches may enable automated optimization of force field parameters guided by information-theoretic targets.

In summary, this work demonstrates that information theory provides a rigorous, quantitative foundation for understanding how molecular-level parameterization choices propagate to macroscopic properties. For researchers selecting water models for molecular simulations, our results strongly support the use of SPC/ε when accurate electronic structure representation is important, while highlighting the fundamental limitations of simpler models like TIP3P for applications requiring realistic water behavior.

## Figures and Tables

**Table 1:** Force field parameters of three-site water models.

Water Model	$r_{\mathrm{OH}}$ (Å)	θ (°)	$q_{H}$ (e)	$q_{O}$ (e)	$\sigma_{\mathrm{OO}}$ (Å)	$\varepsilon_{\mathrm{OO}}/k_{B}$ (K)
TIP3P	0.9572	104.52	+0.417	−0.834	3.1506	76.54
SPC	1.0	109.45	+0.410	−0.820	3.1660	78.20
SPC/ε	1.0	109.45	+0.445	−0.890	3.1785	84.90

**Table 2:** Calculated properties for three water models at 298.15 K and 1 bar.

Property	SPC	SPC/ε	TIP3P	Experimental
Density (kg/m^3^)	977	998	987	998.5 [32]
Dielectric Constant	66.2	78.1	100.2	78.4 [33]
Dipole Moment (Debye)	2.27	2.47	2.35	2.95 [34]
Self-Diffusion Coeff.	3.89	1.48	5.57	2.30 [35]
($\times 10^{-5}$ cm^2^/s)				

## Data Availability

All the data presented in this study is available in the Supplementary Materials.

## Supplementary Materials

The following supporting information can be downloaded at https://www.mdpi.com/article/10.3390/e27101073/s1.
